# Left Infrapatellar Branch of the Saphenous Peripheral Nerve Stimulation Relieves Refractory Pain Following Total Knee Replacement

**DOI:** 10.7759/cureus.67223

**Published:** 2024-08-19

**Authors:** Abigayle M Castine, Christopher L Robinson, Richard N Fair, Giustino Varrassi, Sahar Shekoohi, Alan D Kaye

**Affiliations:** 1 College of Medicine, Louisiana State University Health Sciences Center, Shreveport, USA; 2 Anesthesiology, Critical Care, and Pain Medicine, Beth Israel Deaconess Medical Center, Harvard Medical School, Boston, USA; 3 Anesthesiology, Louisiana State University Health Sciences Center, Shreveport, USA; 4 Pain Medicine, Paolo Procacci Foundation, Rome, ITA

**Keywords:** sedation, peripheral nerve stimulator, neuromodulation, refractory pain, arthroplasty, total knee replacement, osteoarthritis

## Abstract

Osteoarthritis, the most common joint disease of adults worldwide, is increasing in prevalence due to an increase in aging and rates of obesity in developed countries. Treatment options include physical therapy, pharmacologic management, non-pharmacologic management, and total knee replacement surgery. When conservative measures fail, total knee replacement surgery is pursued. The patient is a 61-year-old woman with a history of severe chronic osteoarthritic knee pain following total left and right knee arthroplasty in 2016 and 2019, respectively, who presents with refractory post-total knee replacement pain. Following her surgeries, the patient was in excruciating 10/10 pain on the numerical rating scale (NRS) and was unable to walk or stand. She underwent revisions which, unfortunately, did not ameliorate her pain. She was later referred to chronic pain management in which a peripheral nerve stimulator (PNS) was offered and implanted. Following her PNS trial, the patient achieved >80% pain relief in her left knee. After the permanent PNS implant, the patient noted she had 100% pain relief (0/10 on the NRS) in her left knee and was able to regain mobility. Here, we discuss a case demonstrating rapid pain relief following the minimally invasive PNS implantation for refractory pain following total knee arthroplasty. Refractory pain following total knee arthroplasty can increase morbidity and mortality as a consequence. Thus, proper management is needed to reduce these adverse outcomes. In patients who have failed conservative medical management, PNS may be an alternative, efficacious treatment option for refractory knee pain. Despite the efficacy in our case, further research is needed to define the optimal patient group that would benefit from PNS for refractory knee pain following total knee arthroplasty.

## Introduction

Total knee arthroplasty is the most common surgery conducted to relieve pain caused by osteoarthritis (OA) with 94-97% of operations due to a diagnosis of OA [1,2]. The main indication for surgery is persistent pain at night or with weight bearing [1,2]. With an increase in aging and rates of obesity in developed countries, the percentage of knee replacements has also increased, amounting to 1.5 million operations completed each year globally [3]. In the US alone, the rate of replacements has increased from 31.2 (1970s) to 220.9 (2008) per 100,000 people [1]. Rates amongst women have also increased from 43 (1991) to 137 (2006) per 100,000 women in the UK [1]. Potential risk factors for OA include abnormal loading of joints, age >50, diet, gender (male), genetics, malalignment of joints, obesity, repetitive use, sedentary lifestyle, and trauma/injury [4,5]. Multiple treatments exist for managing pain due to OA, with management being chosen based on symptom severity and duration. Management options include pharmacologic, non-pharmacologic, surgical arthroplasty, or alternative interventions such as implantable peripheral nerve stimulation (PNS) devices [4]. Total knee arthroplasty is often the treatment of choice for individuals with severe symptomatic OA who have failed conservative therapies, yielding a high success rate with significant alleviation of pain and functional capacity [4]. In some cases, intractable pain persists despite total knee arthroplasty, either from the surgery itself or from the original condition. Pain after total knee replacement can be managed with medications, peripheral nerve blocks, physical therapy, additional surgical interventions, dorsal root ganglion stimulation, or implanted PNS, which have been used for decades to offer pain relief for various chronic pain conditions with a recent increase in use in the treatment of refractory post-total knee replacement pain [6].

Persistent chronic pain following total knee replacement is present in a significant number of individuals, with a prevalence of ≥20% 3 to 24 months following surgery [7]. Various observational studies have indicated subgroups in the general population who may be at increased risk for post-operative chronic pain [8]. These groups include female sex, young age (<60) at the time of surgery, greater pre-operative pain level, and psychological influences such as anxiety or depression [7,8]. This case report demonstrates the technical aspects, improved quality of life, safety, and efficacy of PNS implantation for pain relief in refractory post-total knee replacement pain. The exact mechanism of analgesia from PNS is not fully understood but is proposed to be similar in principle to that of transcutaneous electrical nerve stimulation and acupuncture, with an underlying mechanism related to the gate control theory [9]. In 1965, Melzack and Wall proposed the gate control theory, stating that stimulation of large A-beta peripheral nerves modulates pain fiber transmission by small nociceptive afferents, thus reducing pain perception [10].

PNS functions by direct stimulation of a peripheral nerve via subcutaneous implantation of an electrode or lead under sterile conditions with fluoroscopic guidance. The electrode is placed adjacent to the nerve of interest either via direct exposure or through subcutaneous tunneling adjacent to the desired nerve’s course [11]. Anchoring of the electrode then prevents migration or displacement of the leads [11]. The external stimulation system is then programmed to achieve pain control. Related to the need to communicate with the patient while stimulating the affected infrapatellar nerve, sedation rather than general anesthesia is employed, along with local anesthesia [11]. PNS, as a treatment for intractable pain, is an alternative choice due to its effectiveness, low invasiveness, customizable, and quick removal if complications arise [11]. Furthermore, PNS implantation is a generally safe procedure with rapid patient recovery and low morbidity.

## Case presentation

**Figure 1:**
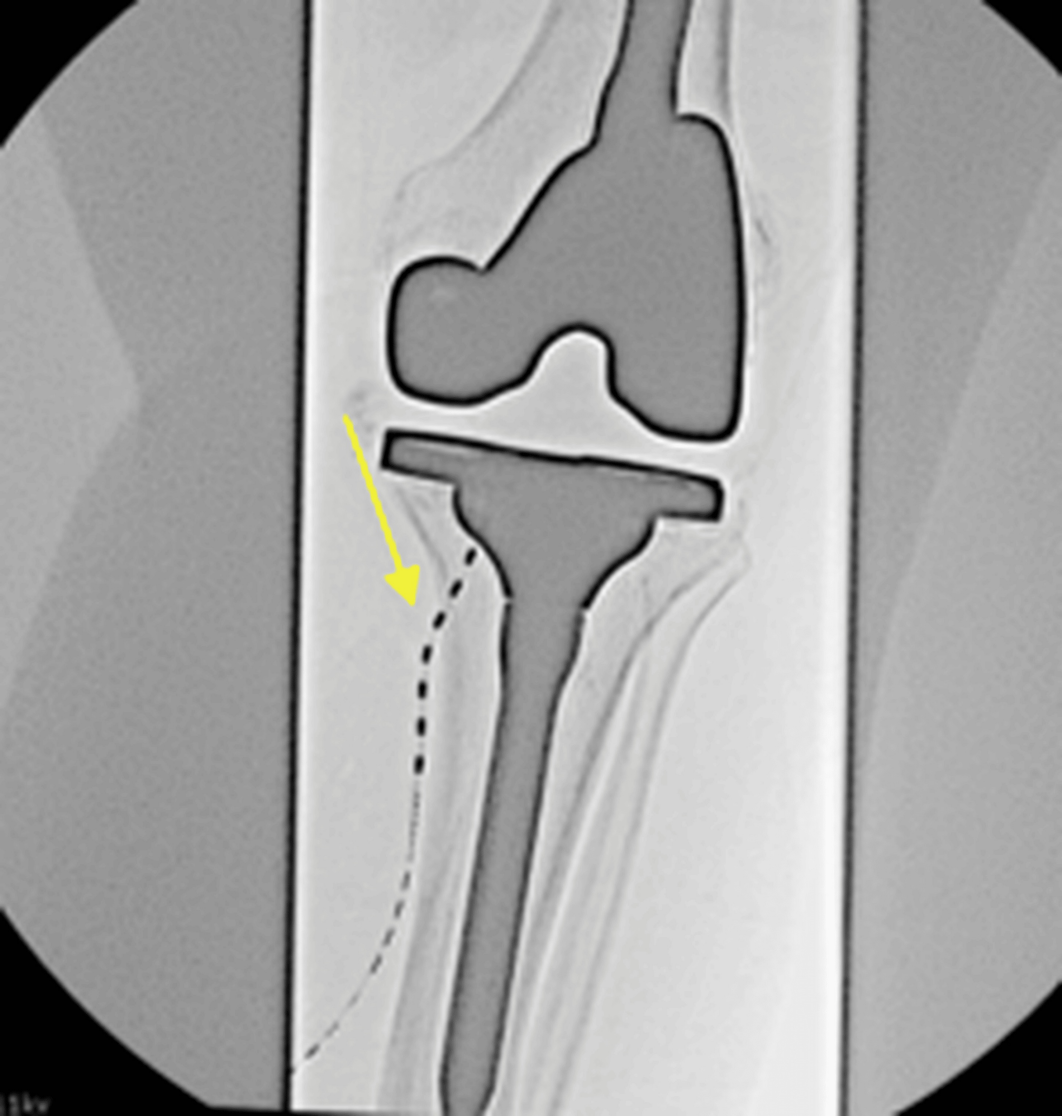
Anteroposterior radiograph of the peripheral nerve stimulator (PNS) trial targeting the left inferomedial genicular nerve. The lead (indicated by the yellow arrow) is positioned along the tibial midshaft and flare.

**Figure 2:**
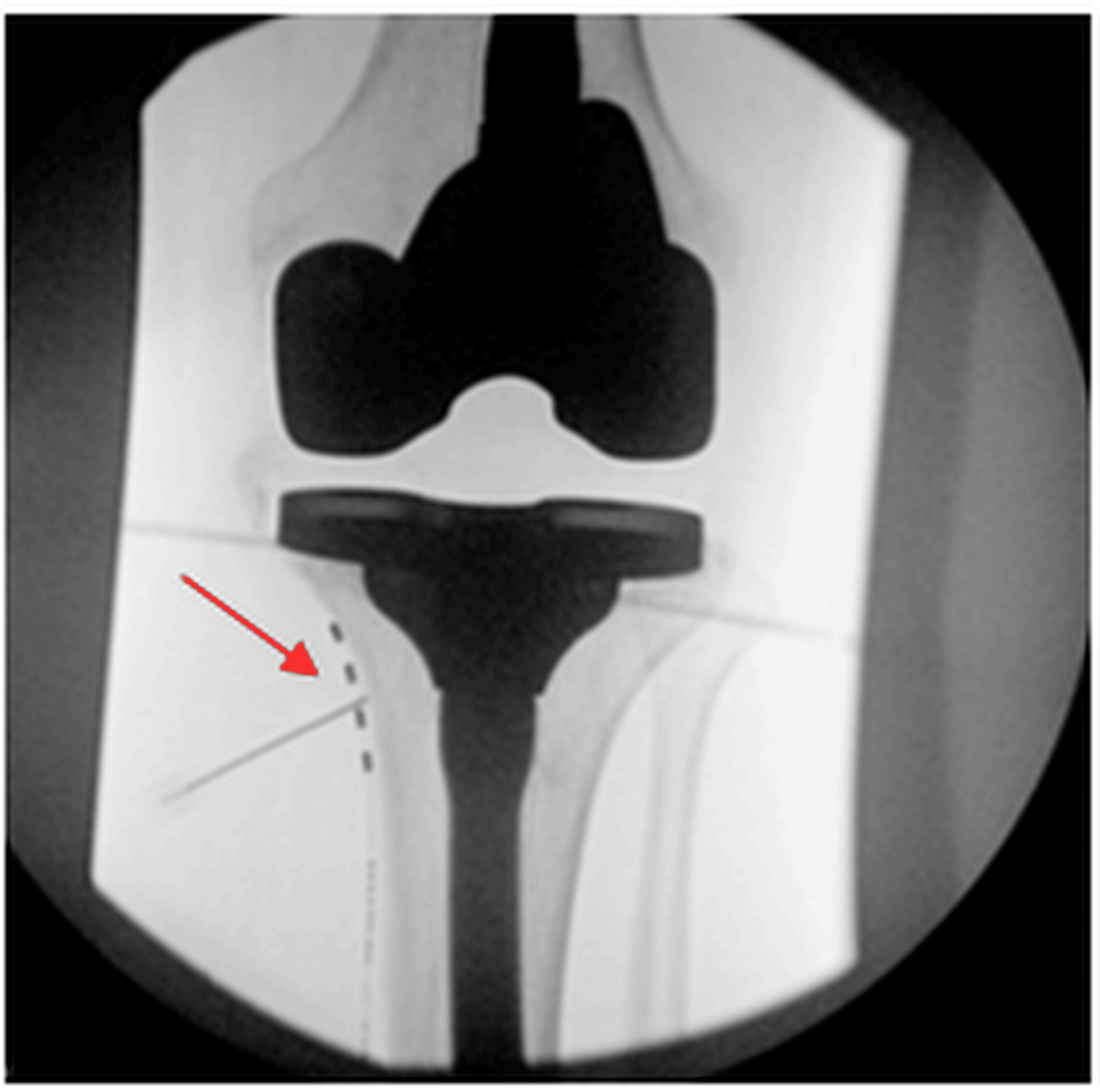
Anteroposterior radiograph of the permanent placement of the peripheral nerve stimulator (PNS) targeting the left inferomedial genicular nerve. The lead (indicated by the red arrow) is positioned along the tibial midshaft and flare.

Our patient is a 61-year-old woman with a past medical history of severe chronic osteoarthritic knee pain status post left and right total knee replacement in 2016 and 2019, respectively, who presents with refractory post-total knee replacement pain. Following the surgeries, she was unable to walk, stand, or work without experiencing significant 10/10 pain, both at rest and during load-bearing activities, which rendered her bed-bound for years. In 2021, she underwent left total knee revision for aseptic loosening of hardware, followed by physical therapy for four months, which did not aid in pain relief. With the persistence of her pain and no further orthopedic interventions offered, she was ultimately referred to pain management. Since the patient failed conservative medical management, a bilateral infrapatellar branch of the saphenous nerve block was performed, providing pain relief in the right but not the left knee. A second left infrapatellar branch of the saphenous nerve block was performed but failed to ameliorate her pain. Before the nerve stimulator implantation procedure, her Oswestry Disability Index score was consistent with complete disability due to lack of relief from painkillers, remaining in bed all day, lack of social life, inability to stand, sleep, lift, or carry, travel, and complete household chores. Finally, a PNS trial was performed targeting her left infrapatellar branch of the saphenous nerve, resulting in >80% pain relief, and a permanent PNS was later implanted (Figures 1-2; see below for procedural details).

The patient was brought to the operating room, administered antibiotics, and placed in the supine position with her left leg propped on a wedge and in slight external rotation. The proximal tibial tuberosity and distal tibia were identified and marked with a permanent marker. After routine sterile preparation, monitored anesthesia care (MAC) was initiated to insert a peripheral nerve neurostimulator device in the left knee. Local anesthetic was injected into the skin overlying the tibial flair along the mid-shaft. The Quincke needle was inserted under fluoroscopic guidance to the tibial flare midshaft. The electrode stimulator was laid across the tibia to determine the incision point on the medial distal left lower extremity. A local anesthetic was administered to the skin, and a small incision was created for the incision point. An incision was made 7-8 cm from the Quincke needle site. Coudé needle was inserted in the distal incision and guided along the tibial midshaft using intermittent fluoroscopy to the target tibial flair marked by the Quincke needle. The Quincke needle was then removed. A nerve stimulator was then placed through a Coudé needle and advanced until the leads were flush with the tibial flair, confirmed with fluoroscopy. The Coudé needle was removed once the stimulator was confirmed to be in the appropriate location with leads on the tibial flair. The stimulator remained in place. Fluoroscopy confirmed that the stimulator remained in the correct place after the Coudé needle was removed. After the injection of local anesthetic, a second incision was made 10 cm distal to the initial incision to insert the coiled distal end of the stimulator. The representative from the nerve stimulator company then tested the leads with a generator using three programs: 2 mA at 1 kHz with a 40 µs pulse width, 2.5 mA at 1499 Hz with a 60 µs pulse width, and 3 mA at 1499 Hz with a 60 µs pulse width and confirmed the patient's responses to these varying stimulations. A tunneler was used to connect incisions to the distal incision in the subcutaneous tissue layer. The distal end of the stimulator was tied, coiled, and sutured to the fascia to prevent uncoiling. Confirmation by fluoroscopy of correct placement was then completed. Hemostasis ensued, and the wound was irrigated with an antibiotic solution. The incision was closed with two-layer sutures, deep and superficial layers, followed by Dermabond (Ethicon, Somerville, USA). The patient tolerated the procedure well and was given a one-week course of antibiotics postoperatively.

After the placement of the permanent PNS, the patient was administered high-frequency stimulation at 1499 Hz. It was determined that she had considerably more relief with the 1499 Hz frequency. She reported significant relief of the left knee pain with a 0/10 visual analog score and the ability to walk, stand, extend, and flex her knee after many years of 10/10 pain that limited her daily activities and range of motion. The parameter for stimulation used in this study is paresthesia-free. The patient’s Oswestry Disability Index score improved to no disability following her procedure, with complete improvement in the ability to stand, walk, lift, bathe, dress, sleep, travel, socialize, and clean without pain.

## Discussion

Following total knee arthroplasty, some patients may have chronic unrelenting pain refractory to conservative measures. Persistent pain is defined as pain lasting for three months or more after total knee arthroplasty and is present in one out of four patients [7]. Many variables have been identified in association with predicting persistent knee pain following total knee arthroplasty. High certainty variables include young age, pain catastrophizing, and moderate-severe acute postoperative pain, with pain catastrophizing having the strongest association - a 23-35% increase in absolute risk [7]. Various studies have attempted to measure and quantify post-surgical and neuropathic pain with few promising treatments due to variations in the prevalence of both factors and the subjectivity of measurement modalities [12]. Both preoperative factors and pain following total knee replacements differ widely among patients, suggesting a more individualized approach be taken in the management of post-surgical pain [12]. A standardized assessment to measure pain severity at post-operative appointments is proposed to improve the management of musculoskeletal pain [12]. An alternative treatment option, when conservative and surgical revisions have been tried, for persistent post-total knee arthroplasty pain is PNS, which has been demonstrated to relieve pain long-term with minimal side effects or complications [13]. The proposed theory of afferent pain fiber inhibition, preventing synapses from being transmitted to the brain, allows for selective and targeted pain modulation and, ultimately, relief with blockage of pain signals [13]. In accordance with recent studies, a significant improvement in immediate pain relief, quality of life, and satisfaction has been demonstrated when used to treat post-surgical pain [6,14]. Moreover, a randomized, double-masked, multicenter study demonstrated long-term pain relief as a result of PNS implantation [15]. In this regard, PNS is less invasive with associated complications than dorsal root ganglion stimulation or spinal cord stimulation [16]. Our case report provides another example of utilizing PNS in chronic, unrelenting pain in a post-total knee arthroplasty patient who has failed conservative and invasive management. Via a minimally invasive procedure, PNS offered maximal pain control and allowed our patient to have restoration of range of motion and the ability to walk and stand again, the latter two of which caused unbearable pain just a week prior to the implant.

## Conclusions

Our case report provides another example of utilizing PNS in chronic, unrelenting pain in post-total knee arthroplasty patients who have failed conservative and invasive management. Via a minimally invasive procedure, PNS offered maximal pain control and allowed our patient to restore range of motion and the ability to walk and stand again, the latter two of which caused unbearable pain just a week prior to the implant. Further studies are needed to investigate the long-term efficacy of implanted PNS devices on persistent post-total knee arthroplasty pain.
